# Supplementary material for: FastqPuri: high-performance preprocessing of RNA-seq data
Source: BMC Bioinformatics. 2019 May 3;20:226. doi: 10.1186/s12859-019-2799-0 (PMC6500068; doi:10.1186/s12859-019-2799-0)
Supplement: Supplementary file 2 — Archive of FastqPuri. Archive containing all files needed to install and run FastqPuri v1.0.6. Date stamp March 22, 2019. (GZ 47,819 kb) [file 12859_2019_2799_MOESM2_ESM.gz › FastqPuri-1.0.6/html/md_README_Sreport.html]

My Project: SReport user manual


My Project

SReport user manual

Creates two types of summary reports in html format

- **Q** (Quality): report of the quality status of a set of `fastq` files. Needs the binary output of `Qreport`, for a list of files. It will analyze all files with extension `*.bin` inside the folder specified in the input and will expect them to have the appropriate format (output from `Qreport`).
- **F** (Filter): report of the filtering process (`trimFilter`) of a set of `fastq` files. It will analyze all files with extension `*bin` inside the folder specified in the input and will expect them to have the appropriate format, (output from `trimFilter`).
- **D** (Filter): report of the filtering process (`trimFilterDS`) of a set of double stranded `fastq` files. It will analyze all files with extension `*bin` inside the folder specified in the input and will expect them to have the appropriate format, (output from `trimFilterDS`).

## Running the program

Usage `C` executable (in folder `bin`):

Usage: ./Sreport -i <INPUT\_FOLDER> -t <Q|T|D> -o <OUTPUT\_FILE>

Uses all \*bin files found in a folder (output of Qreport|trimFilter)

and generates a summary report in html format (of Qreport|trimFilter).

Options:

-v Prints package version.

-h Prints help dialog.

-i Input folder containing \*bin data (output from Qreport). Mandatory option.

-t {Q,F,D} Type of report to be generated: 'Q' for quality summary

report, 'F' for filter summary report, and 'D' for double stranded

data filter summary report. Mandatory option,

-o Output file (with NO extension). Mandatory option.

## Output description

- **Q** (html output):
  - Table with: `# reads , # tiles, % lowQ reads, % reads with N’s` for all samples.
  - Heatmap showing the average quality per position for all samples.
- **F** (html output):
  - Table with .trimFilter setup. If there were samples with different setups, this table is ambiguous and is therefore not created.
  - Table with : `Nreads`, `Naccepted`, `disc Ad`, `cont`, `disc lowQ`, `disc N’s`, `trim Ad`,`trim N’s`,`trim lowQ` for all samples.
- **D** (html output):
  - Table with .trimFilterDS setup. If there were samples with different setups, this table is ambiguous and is therefore not created.
  - Table with : `Nreads`, `Naccepted`, `disc Ad`, `cont`, `disc lowQ`, `disc N’s`, `trim Ad`,`trim1 N’s`,`trim1 lowQ`, `trim2 N’s`, `trim2 lowQ` for all samples.

## Example

### Option `-t Q`

An example is given in the folder `examples/QReport_Sreport`. To run an example, type,

$ cd example/Qreport\_Sreport/run\_test/

$ ../../../bin/SReport -i ./ -t Q -o my\_test\_summary\_report

and compare it with the provided run example, as specified in the README file under `./examples/QReport_Sreport`

**NOTE:** it has to be run AFTER `Qreport` example

### Option `-t F`

In folder `.examples/trimFilter_Sreport/bin_files`, 30 fake `./trimFilter` binary output files were generated (with the `R` script `create_fake_bins.R`). An html output was created out of them (`.examples/trimFilter_Sreport/bin_files/filter_Sreport_example.html`). It can be reproduced if you run:

$ ../../bin/Sreport -i .bin\_files/ -o ./bin\_files/filter\_Sreport\_new -t F

### Option `-t D`

In folder `.examples/trimFilterDS_Sreport/bin_files`, 30 fake `./trimFilterDS` binary output files were generated (with the `R` script `create_fake_bins.R`). An html output was created out of them (`.examples/trimFilterDS_Sreport/bin_files/DS_Sreport_example.html`). It can be reproduced if you run:

$ ../../bin/Sreport -i .bin\_files/ -o ./bin\_files/DS\_Sreport\_new -t F

## Contributors

Paula Pérez Rubio

## License

GPL v3 (see LICENSE.txt)


---

Generated by  

 1.8.14
